# Parasitised caterpillars suffer reduced predation: potential implications for intra-guild predation

**DOI:** 10.1038/srep42636

**Published:** 2017-02-23

**Authors:** Wen-bin Chen, Liette Vasseur, Min-sheng You, Jian-yu Li, Cheng-xiang Wang, Ruo-xue Meng, Geoff M. Gurr

**Affiliations:** 1 Institute of Applied Ecology and Research Centre for Biodiversity and Eco-Safety, Fujian Agriculture and Forestry University, Fuzhou, China; 2 State Key Laboratory of Ecological Pest Control for Fujian and Taiwan Crops, Fuzhou, China; 3 Department of Biological Sciences, Brock University, St. Catharines, Ontario, Canada; 4 Graham Centre, Charles Sturt University, Orange, New South Wales, Australia

## Abstract

Intra-guild predation (IGP) is an important phenomenon structuring ecological communities and affects the success of biological control. Here we show that parasitism by the koinobiont wasp *Cotesia vestalis* is associated with behavioural changes in its larval host (diamondback moth, *Plutella xylostella*) that reduce risk of IGP. Compared with unparasitised caterpillars, parasitised *P. xylostella* moved less frequently to new feeding patches on plants and were less likely to fall from the plant. Wolf spiders killed significantly fewer parasitised larvae. Reflecting their reduced movement and capacity to select plant tissue of optimal quality, parasitised caterpillars fed at a lower rate and exhibited delayed development suggesting a trade-off between IGP avoidance and nutrient intake by the host. This change in behaviour to reduce risk may cascade to the first trophic level and help explain the stability of IGP systems.

There are many reports of parasites manipulating the behaviour of their host^1^,^2^,^3^,^4^,^5^,^6^,^7^,^8^. Among the best studied cases of this puppet master phenomenon^9^ are those where the host is manipulated to perform bizarre, abnormal behaviour^10^, or where the manipulation of the ‘zombie’^11^ host culminates in its grotesque death. In such cases, altered host behaviour is associated with facilitating the parasite life cycle^12^,^13^. For example, the nematomorph *Paragordius tricuspidatus* Dufour causes its cricket host (*Nemobius sylvestris* Bosc D’Antic) to jump suicidally into water^4^, whilst infection of gypsy moth (*Lymantria dispar* L.) by baculoviruses result in the larva climbing to the top of trees to die^14^, resulting in benefit to the parasite by enhanced dissemination or host location. Underling this phenomenon, parasites have been reported to have effects on host development, physiology and immune system^15^,^16^,^17^.

Koinobiont parasitoids such as *Cotesia vestalis* (Haliday) (Hymenoptera: Braconidae) allow the host to continue feeding and development while feeding upon it; in contrast to “idiobionts” in which host development ceases once parasitised. This is an important life history strategy for the parasitoid to ‘capture’ the host at early stage yet allowing it to feed to compensate for its initially low nutritional value^18^. If, however, the host is killed before the parasitoid has developed sufficiently, the parasitoid perishes too^19^. Reflecting these facts, koinobionts can be predicted to avoid disrupting the host’s normal, evolutionarily-optimised feeding behaviour unless there is a sufficiently large gain by reducing risk from mortality.

Intra-guild predation (IGP), where competing species prey on each other as well as on a shared prey^20^,^21^, has emerged as a common phenomenon in ecological communities^22^. A predator and a parasitoid can attack a shared prey/host species^23^ but the resulting IGP is asymmetrical because, whilst the predator can consume parasitised hosts, the parasitoid cannot exert reciprocal attack on the predator. Parasitoid adults can alter their foraging to reduce the risk of IGP^24^ and immobile parasitised aphid ‘mummies’ can develop a thickened, protective cuticle to prevent attack^25^. It is, however, unclear if the capacity of immature koinobiont parasitoids extends to altering the behaviour of their active hosts to minimise IGP risk.

The overarching aim of this study was to test the hypothesis that the larval foraging behaviour of the globally significant brassica pest, diamondback moth, *Plutella xylostella* L. (Lepidoptera: Plutellidae), is altered when parasitised by *C. vestalis* in a manner that reduces the risk of IGP. Movement on a plant is risky for insect herbivores^26^,^27^,^28^. In particular, herbivores that move to detect superior feeding sites may be exposed to elevated risk from predators^29^. Accordingly, an initial study tested the hypothesis that parasitized hosts exhibit reduced movement on foliage compared with healthy larvae; whether or not caused by the parasitoid manipulating the behaviour of the host. Any such change in foraging could originate as a ‘side effect’ of parasitism that led to greater lethargy in the host but could still be acted upon by natural selection. Evolution is not directed or teleological so any phenotypic variability in the parasitoid that happens to have an effect on the host and leads it to move less often would increase fitness if it reduced mortality by factors such as intra-guild predation. Though Poulin^2^ proposes multiple criteria to establish whether behavioural modifications may be considered as manipulation by parasites, including the complexity of the behavioural change, these were subsequently considered to be too conservative and that demonstration of fitness benefits provided the strongest evidence of parasite manipulation^30^. Accordingly, potential parasite manipulation can manifest as a change in host behaviour that is modest compared with the more dramatic examples described above. Subsequent studies explored whether fitness was enhanced in our study system using simulated wind or foraging *C. vestalis* since both these types of disturbances are faced by *P. xylostella* in the field; whilst predation was measured in the presence of the wolf spider (*Pardosa pseudoannulata* Koch (Araneae: Lycosidae). Each of these experiments tested the hypothesis that parasitized larvae were less strongly affected by the respective disturbance factor than were healthy hosts. Finally, we tested the hypothesis that reduced movement on the plant by parasitized larvae results in a reduction in nutrient acquisition by parasitised larvae, consistent with a trade-off from the altered feeding behaviour leading to a poorer quality diet.

## Results and Discussion

**Figure 1:**
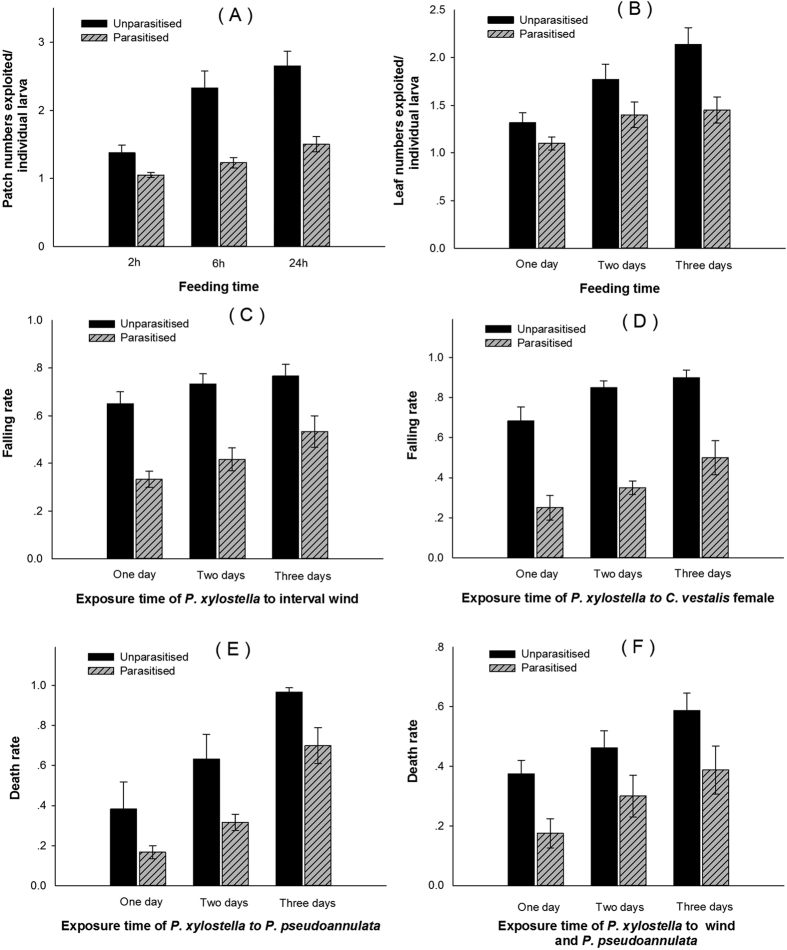
Effect of parasitism on feeding behaviour and fate of *P. xylostella* larvae. (**A**) Numbers of feeding patches exploited on a single leaf (±SE) (*t-*test, 2 h: t = −2.924, df = 47.429, p = 0.005; 6 h: t = −4.225, df = 46.172, p < 0.001; 24 h: t = −4.707, df = 58.854, p < 0.001) (**B**) Numbers of separate leaves exploited (±SE) (Treatment main effect: F = 6.612, p = 0.013; Time main effect: F = 40.518, p < 0.001; Treatment * Time: F = 4.199, p* = *0.028). (**C**) Proportion of larvae falling from plant under the influence of simulated wind (±SE) (Treatment main effect: F = 26.1, p < 0.001; Time main effect: F = 10.112, p = 0.001; Treatment * Time: F = 0.933, p = 0.410); (**D**) Proportion of larvae falling from plant in presence of a foraging *C. vestalis* female (±SE) (Treatment main effect: F = 44.693, p < 0.001; Time main effect: F = 16.818, p < 0.001; Treatment * Time: F = 0.795, p = 0.465); (**E**) Proportional mortality in presence of a predator (*P. pseudoannulata*) allowed to forage on the plant (±SE) (Treatment main effect: F = 7.569, p = 0.020; Time main effect: F = 39.160, p < 0.001; Treatment * Time: F = 0.305, p = 0.740); (**F**) Proportional mortality under the influence of simulated wind and in presence of a predator (*P. pseudoannulata*) prevented from foraging on the plant (±SE) (Treatment main effect: F = 6.081, p = 0.027; Time main effect: F = 17.144, p < 0.001; Treatment * Time: F = 0.178, p = 0.838).

Parasitised larvae exploited significantly (p < 0.005) fewer feeding patches on excised leaves compared with unparasitised larvae in 2, 6 and 24 hour-long experiments (Fig. 1A). Similarly, reduced movement of parasitised larvae was evident in a separate study using whole plants that extended over three successive days and assessed the numbers of separate leaves fed upon by individual larvae (GLM treatment main effect, F = 6.612, p = 0.013; Fig. 1B). Subsequent studies tested whether this reduced host movement might be adaptive for the immature parasitoid by reducing the risk of IGP.

In a study in which a light breeze was simulated using an electric fan, parasitised larvae had a significantly lower rate of falling from whole plants compared with unparasitised larvae (GLM treatment main effect, F = 26.1, p < 0.001; Fig. 1C). In a similar study in which wind was replaced by a gravid *C. vestalis*, parasitised larvae were again much less likely to fall from the plant than were unparasitised larvae (GLM treatment main effect: F = 44.693, p < 0.001; Fig. 1D). This study did not partition the potential effect of the foraging parasitoid discriminating against parasitised larvae and focusing greater attention on unparasitised hosts rather than engaging in superparasitism. Any such effect is, however, likely to be minor because once gravid *Cotesia* detect an infested plant the formerly important orientation by chemical cues associated with herbivory^31^ is replaced by random searching until antennal contact with the host^32^. Observations of parasitoid foraging made during our study were consistent with this non-selective host location scenario. Accordingly, the higher rate of dropping from the plant by unparasitised hosts reflects response of these larvae rather than their detectability.

A further study used single wolf spiders as intra-guild predators. *Pardosa* species, also known as ground spiders^33^, are generally considered ground-foraging, active hunters^34^ but also forage on low foliage^35^. Whilst spiders consumed nearly all of the unparasitised larvae in our three-day-long mesocosms studies, significantly fewer of the parasitized larvae were eaten (GLM treatment main effect: F = 7.569, p = 0.020; Fig. 1E). A supplementary study without plants established that spiders did not discriminate between parasitised and unparasitised larvae and that equal numbers of each were consumed (Supplementary Figure 1) thus demonstrating the significance of on-plant behaviour and dislodgement in relative predation risk of parasitised and unparasitised larvae.

An additional study confined wolf spiders to the floor of mesocosms by mounting the plant atop a glass column up which only the larvae were able to climb. By confining the spiders to the floor of the arena, this removed the effect of spiders directly disturbing caterpillars and causing them to fall from the plant. Accordingly, only caterpillars that fell as a result of the simulated light breeze became available as prey. Simulated wind was applied as previously described and dislodged larvae were exposed to predation. Under these conditions, parasitised larvae were significantly less likely to be consumed (GLM treatment main effect, F = 6.081, p = 0.027; Fig. 1F).

Predator avoidance is recognised to constrain Lepidoptera larvae foraging to less than the nutritionally and physiologically ideal diets^29^,^36^. For example, herbivory in the field has been shown to be reduced by the presence of predators^37^. Patch feeding is employed by herbivores, including *P. xylostella*^38^, to locate plant tissue of optimal nutritional value and this can involve avoidance of sites in which plant defences have reduced the local quality of the plant tissue. Though *P. xylostella* is adapted to *Brassica* spp. allelochemicals such as glucosinolates, reduced movement of larvae may result in consumption of plant tissue that is harder, waxier, and lower in nutritional value^27^.

**Figure 2:**
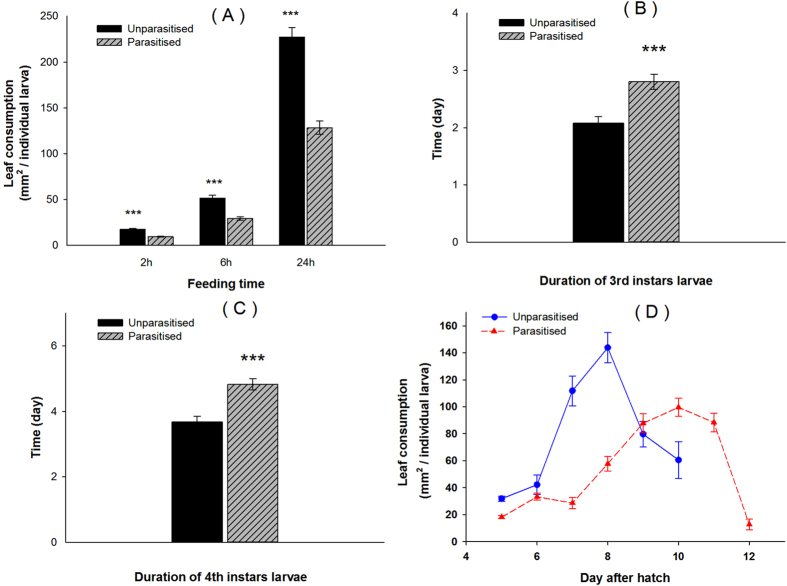
Effect of parasitism on feeding and development of *P. xylostella* larvae. (**A**) Short-term leaf consumption (±SE) of parasitised and unparasitised larvae on cabbage (*t*-test, 2 h: t = −7.412, df = 61.743, p < 0.001; 6 h: t = −6.639, df = 65.755, p < 0.001; 24 h: t = −7.858, df = 78, p < 0.001); (**B**) Duration (±SE) of parasitised and unparasitised 3rd instar *P. xylostella* larvae; (**C**) Duration (±SE) of parasitised and unparasitised 4th instar *P. xylostella* larvae; (**D**) Daily leaf consumption (±SE) of parasitised and unparasitised larvae (*t*-test, day5: t = −6.044, df = 65.155, p < 0.001; day 6: t = −1.067, df = 46.047, p = 0.291; day 7: t = −6.992, df = 48.237, p < 0.001;. day 8: t = −6.858, df = 53.856, p < 0.001; day 9: t = 0.292, df = 67, p = 0.771; day 10: t = 2.568, df = 29.627, p = 0.016.

Our results showed that parasitised *P. xylostella* larvae moved less frequently on cabbage foliage than unparasitised larvae and this was associated with reduced risk of falling from the plant and IGP but this appears to carry a trade-off in terms of nutrient acquisition. In the study of patch movement on excised leaves, parasitised larvae consumed less than unparasitised counterparts (GLM treatment main effect: F = 47.421, p < 0.001) (Fig. 2A). This can be attributed to the larvae handling poorer quality plant tissue because parasitism did not affect the total duration of pauses in feeding or the number of feeding interruptions (Supplementary file, Supplementary Figures 2 and 3). Our longer term study using intact plants found that larval development was slower for parasitised compared with unparasitised *P. xylostella (t*-test, 3rd instars: t = 4.083, df = 78, p < 0.001; 4th instars: t = 4.747, df = 71, p < 0.001) (Fig. 2B,C), an effect in agreement with Shi, *et al*.^39^ and Bae and Kim^17^. Daily leaf consumption on whole plants peaked at 143.84 ± 11.14 mm^2^ on day 8 for unparasitised larvae but parasitised larvae consumed no more than 99.58 ± 6.66 mm^2^ per day with this peak not occurring until day 10 (Fig. 2D). The extended developmental time of parasitised larvae meant that total leaf consumption during development was similar to that of unparasitised larvae, 384.38 ± 24.62 mm^2^ versus 442.99 ± 20.70 mm^2^, respectively (*t*-test, t = −1.822, df = 78, p = 0.072). This reduced rate of nutrient acquisition may represent a trade-off for reduced risk of IGP. Such a scenario is consistent with increased fitness of the parasitoid provided that the disadvantages of slowed development are more than offset by a reduced risk of dislodgement and predation, with natural selection acting to favour the trade-off ‘setting’ that optimises fitness.

Overall these results are consistent with, rather than conclusive evidence for, parasitoid manipulation of host behaviour in this system. If confirmed in future work, this provides a formerly unrecognised form of parasite manipulation that has potential cascading effects to the first trophic level. Though parasitised larvae fed on similar volumes of plant tissue, their diet is likely to be poorer quality and less valuable to the plant than that consumed by more mobile unparasitised larvae. Further, the diet of parasitised larvae is consumed over a longer time period, better allowing the plant to undergo compensatory growth. The ecological significance of cascading effects on the first trophic level from this form of parasite manipulation may be important, especially in agroecosystems, but remains to be explored. Clearly, field studies to test for the strength of this phenomenon under more realistic and complex conditions are necessary.

*Cotesis vestalis* has been reported to be an effective biological control agent of *P. xylostella* in multiple countries^40^,^41^,^42^. If changes in parasitized host behaviour are confirmed in field studies, it may help explain the success of this species and signal the need for equivalent work in other host-parasitoid systems including those of biological control significance.

Fire ants (*Solenopsis invitica* (Boisduval)) parasitised by the decapitating fly (*Pseudacteon tricuspis* Borgmeier) have been reported to remain in the nest rather than engaging in foraging^11^ and this may reflect manipulation of host behaviour to reduce predation risk to the parasitoid. In that study, however, there was no assessment of IGP and no trade-off in nutrient acquisition because the parasitoid consumes only the head capsule contents rather than the entire host as is the case with *Cotesisa*. Because IGP exerts such negative effects on parasitoids, any available trait that reduces risk is likely to be selected^24^. Accordingly, even a minor change in host behaviour, such as reduced movement that may originate as a ‘side effect’ of parasitism, potentially provides a fitness advantage to the parasitoid. Our studies appear to be the first to test for effects consistent with the behaviour of a parasitised host being manipulated by its parasitoid in order to reduce IGP. If this phenomenon is found to occur in the field and within multiple host-parasite systems it would help reconcile the theoretical instability of these tri-partite systems^20^ with the fact that they are ubiquitous in nature^22^. Complexity appears key to developing robust models of IGP^43^ and parasitoid puppet masters might play a formerly-unrecognised stabilising role.

## Materials and Methods

### Insect and plant preparation

Founders for the *P. xylostella* and *C. vestalis* cultures were sourced as pupae/cocoons from a cabbage field in the town of Nantong, Fujian Province, in May 2014. The spider, *P. pseudoannulata*, was from a colony at the Institute of Plant Protection, Fujian Academy of Agricultural Sciences. All arthropods were maintained and tested in an environmental chamber at 25 ± 2 °C, 50% ± 10% RH, with a photoperiod of L14:D10. *P. xylostella* was reared on radish sprouts (*Raphanus sativus* L.) and *C. vestalis* was reared on larvae of *P. xylostella*. To prepare parasitised *P. xylostella*, neonate larvae were placed on cabbage for five days then third instar larvae were randomly chosen. Each larva was exposed to a mated female *C. vestalis* individually in a glass tube and monitored until parasitism occurred, a protocol that has been shown to lead to a parasitism rate of over 93%^44^.

Cabbage (*Brassica oleracea* var. *capitata* (L.), cv. Jing-feng No.1) was sown in boxes (40 cm × 30 cm × 14 cm) and placed in an outdoor mesh cage. About 20–30 days after seeding (2–3 leaf stage), individual plants were transplanted in larger pots (9 cm diameter). The cabbage plants were at the 4–6 leaf stage when used in the experiments.

### Larval feeding patch assessment and short-term leaf consumption

To investigate the movement of larvae from patch to patch on a leaf, parasitised and unparasitised 3^rd^ instar larvae were individually reared on the cabbage until they reached the 4^th^ instar. Larvae were starved for 6 hours before being introduced individually to a fresh cabbage plant. The larvae were then allowed to feed for 2 hours and the numbers of feeding patches the larvae exploited were recorded for forty replicates. This was repeated for periods of 6 and 24 hours feeding with different larvae and plants. The leaf area consumed was measured using a calibrated stereomicroscope (Olympus-SZX2) and analysed using the software “cellSens dimension” (Olympus Ltd, Tokyo, Janpan) to determine leaf consumption by each larva. Student’s *t*-test was used to compare unparasitised and parasitised larvae wihin each of the three experiments.

### Larval movement from leaf to leaf

Thirty parasitised and thirty unparasitised 3^rd^ instar larvae were individually placed on the third leaf of intact cabbage plants. The numbers of cabbage leaves fed upon by each larva were recorded every day for three days. A General Linear Model (GLM) with repeated measures was used to determine the effect of treatments (between-subject variation) and time (within-subject variation). The measures were the numbers of leaves fed upon, with data being collected on successive time periods.

### Effect of simulated wind on larval dislodgement

To determine the effect of wind on dislodgement from the plant of parasitised versus unparasitised larvae, a system was designed to simulate wind of a nature likely to be encountered in the field. Two potted cabbage plants were placed on a bench equidistant from an oscillating, electric fan with parasitised larvae on one plant and unparasitised larvae on the second. These constituted one block and replication was achieved by use of six such systems. Air speed was measured using a wind meter (testo405-V1 (Testo Ltd, Stuttgart, Germany)) to peak at 1.2 m/s (a ‘light breeze’ in the Beaufort scale) with a periodicity between oscillations of 3 sec. Prior to the fan being turned on, 10 parasitised or unparasitised 3^rd^ instar larvae were placed on the plants to settle for 30 min and during this period, fallen larvae were placed back onto the plant and all individuals were observed to have established feeding. The fan was then turned on and ran continuously for three days. Each day, numbers of larvae trapped on a sticky base beneath each plant were counted. A General Linear Model (GLM) with repeated measures was used to compare the effect of parasitism, time and interaction. The measures were the numbers of fallen larvae, with data being collected for each of these on successive time periods.

### Effect of foraging *C. vestalis* on larval dislodgement

Individual cabbage plants were placed in cuboid plastic arenas (15 cm × 15 cm × 25 cm) with a ventilated mesh lid. Prior to use, plants were removed from soil, the roots wrapped with wet cotton wool (adding water every day) and then covered with silver paper. The base of the arena was covered by a layer of water 8–10 mm deep to trap larvae that fell from the plants. Ten 3^rd^ instar larvae were used per plant with six replicates for each of parasitised and unparasitised larvae. After larvae settled for 30 min, a single mated *C. vestalis* female was introduced to the arena, and a cotton wool swab soaked in 10% honey solution was put on the mesh lid (adding honey solution every day). Fallen larvae were removed and counted daily for three days. The falling rate (number of larvae falling from the plant) was analysed as described for the simulated wind experiment.

### Effect of foraging spider on larval mortality

This study used plastic arenas (long: 24 cm, wide: 15 cm, tall: 9 cm) with one 4^th^ instar female spider (*P. pseudoannulata*) in each. The spider was previously starved for 48 hours and a wet cotton wool swab was placed in the arena to supply it water. Plants were prepared as described for the *C. vestalis* study with 10 parasitised or unparasitised larvae placed on the plant and allowed to settle for 30 minutes. The spider was placed on the floor of the arena and was free to forage there and over the plant. Larvae remaining on the plant were recorded every 24 hours for three days whilst all larvae that fell from the plant were consumed by the spider. A ring of tacky adhesive around the arena’s wall prevented larvae from leaving but without entrapping them. Both unparasitised and parasitised treatments were repeated eight times. Data were analysed as described for the simulated wind experiment.

### Effect of ground-foraging spider and simulated wind on larval mortality

This study used a mesocosm design similar to the preceding spider study except simulated wind was applied to the plants (as described previously) and spiders were confined to the floor of the arena by placing the plant roots in a glass cylinder (diameter: 2.5 cm, height: 7 cm and filled with water) up which larvae, but not the spider, were able to climb back onto the plant. Ten larvae (parasitised or unparasitised) were used on each plant and numbers remaining on the plant were recorded every 24 hours for three days. All larvae that fell from the plant were consumed by the assessment time each day. Eight replicates were used and data were analysed using GLM as describe above.

### Larval development and long-term leaf consumption

To measure the duration of 3^rd^ and 4^th^ (final) instar stages, 40 parasitised and 40 unparasitised 3^rd^ instars were placed individually on cabbage plants. The leaf bearing the larva was sealed in a punctured plastic bag (10 cm × 7 cm) to prevent escape. After 1 day, each larva was transferred onto a fresh plant, until death or pupation. Damaged leaves were removed from plants to measure leaf consumption as described for the short term consumption study (above). Student’s *t-*test was used to compare the total leaf consumption and development time of unparasitised and parasitised larvae. Since the number of individuals varied from day to day due to pupation and death, to explore where there were significant differences between the two treatments, a *t*-test were performed to compare each day separately instead of a repeated measures analysis of variance.

## Additional Information

**How to cite this article**: Chen, W.- *et al*. Parasitised caterpillars suffer reduced predation: potential implications for intra-guild predation. *Sci. Rep.* **7**, 42636; doi: 10.1038/srep42636 (2017).

**Publisher's note:** Springer Nature remains neutral with regard to jurisdictional claims in published maps and institutional affiliations.

## Supplementary Material

Supplementary Information
