# Supplementary material for: Parasitised caterpillars suffer reduced predation: potential implications for intra-guild predation
Source: Sci Rep. 2017 Feb 23;7:42636. doi: 10.1038/srep42636 (PMC5322372; doi:10.1038/srep42636)
Supplement: Supplementary Information [file srep42636-s1.pdf]

SUPPLEMENTARY MATERIAL

Parasitised caterpillars suffer reduced predation, potential implications for intra-guild predation

Wen-bin Chen[1,2,3,4], Liette Vasseur[1,5], Min-sheng You[1,2,3,4], Jian-yu Li[1,2,3,4], Cheng-xiang Wang[1,2,3,4], Ruo-xue Meng[1,2,3,4], Geoff M. Gurr[1,6]

[1]Institute of Applied Ecology and Research Centre for Biodiversity and Eco-Safety, Fujian Agriculture and Forestry University, Fuzhou, China, [2]Fujian-Taiwan Joint Innovation Centre for Ecological Control of Crop Pests, Fujian Agriculture and Forestry University, Fuzhou, China, [3]Key Laboratory of Integrated Pest Management for Fujian-Taiwan Crops, Ministry of Agriculture, China, Fuzhou, China, [4]Fujian Provincial Key Laboratory of Insect Ecology, Fujian Agriculture and Forestry University, Fuzhou, China, [5]Department of Biological Sciences, Brock University, St. Catharines, Ontario, Canada, [6]Graham Centre, Charles Sturt University, Orange, New South Wales, Australia

Predation by *P. pseudoannulata* of parasitized and unparasitised *P. xylostella* larvae in plant-free arenas

This study used simple, plant free plastic arenas (24 cm long × 15 cm wide × 4 cm tall). The spiders were unable to climb the walls but *P. xylostella* were so a mesh lip prevented these from climbing out of reach of the spiders. Ten parasitised and ten unparasitised larvae were randomly placed in each of four arenas. A small dot of ink was used to mark larvae according

to parasitism status. To avoid introducing a possible effect of the ink, its use to denote either parasitised or unparasitised treatments was random across replicates. Ten minutes after the larvae were placed in the arenas, a single 4th instar female spider (*P. pseudoannulata*) was introduced in each arena. The spider was previously starved for 48h. Numbers of parasitised and unparasitised larvae remaining in the arena were recorded after 20 min, 40 min and 60 min. Repeated measures GLM was used for statistical analysis.

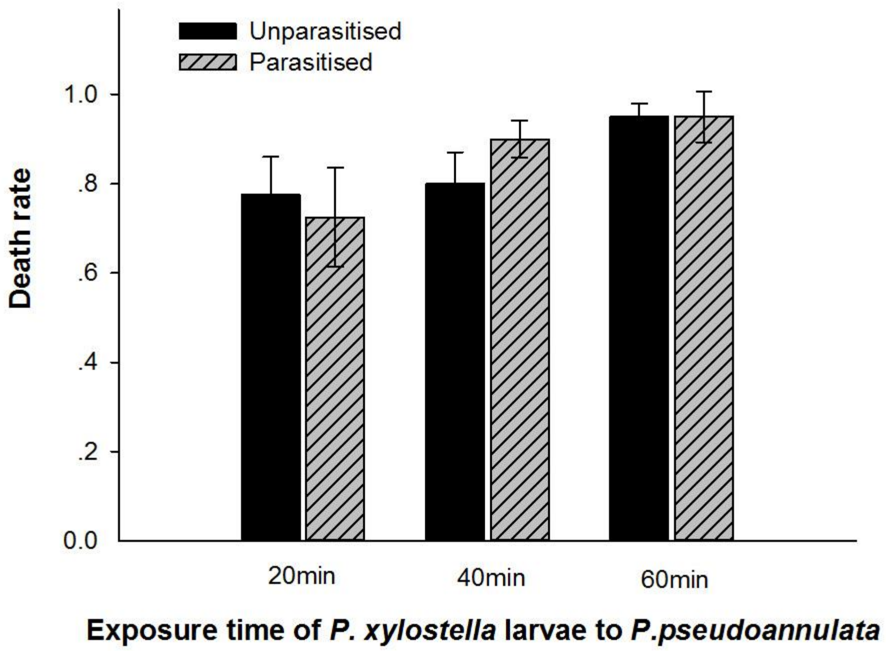

Supplementary Figure 1. Nil effect of parasitism in *P. xylostella* larvae on predation by the spider, *P. pseudoannulata,* in simple, plant-free arenas (Treatment main effect: $F = 0.036$, $p = 0.856$; Time main effect: $F = 12.522$, $p = 0.001$; Treatment * Time: $F = 1.826$, $p = 0.203$).

The spiders showed no significant preference for larvae of either treatment at any of the successive time periods (Supplementary Figure 1).

Feeding behaviour of parasitised and unparasitised *P. xylostella* larvae

Eight parasitised and eight unparasitised 4th instar *P. xylostella* were placed individually on the abaxial surface of excised cabbage leaves in Petri dishes on the platform of an observation chamber with camera and recording system CASO-L (Camsonar Ltd, London, UK). The leaf petiole was wrapped with wet cotton wool to maintain turgidity and the edge of the leaf was secured to the Petri dish with adhesive tape to prevent the larva from accessing the adaxial surface and preventing its behaviour from being monitored. The number and duration feeding periods were recorded for 6 hours. GLM with repeated measures was used to compare parasitised and unparasitised larval behaviour for 2, 4 and 6 hours periods.

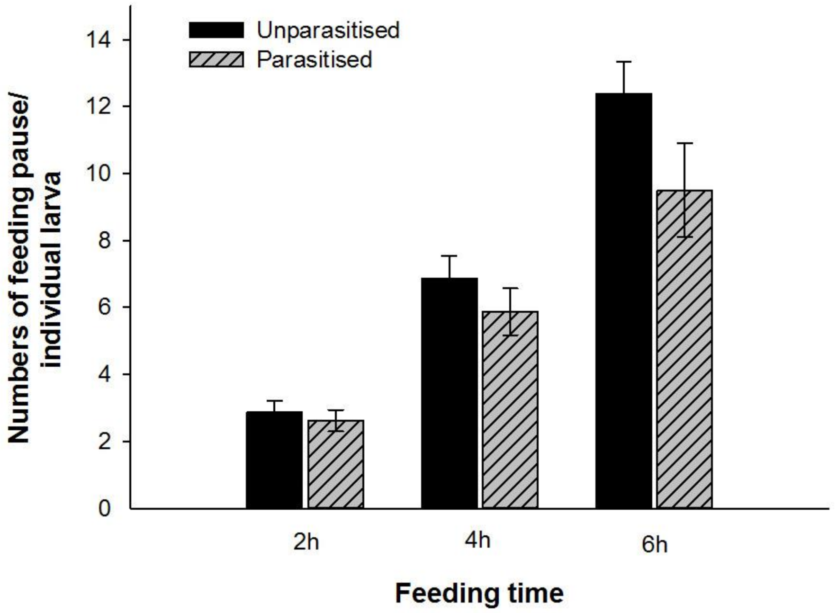

Supplementary Figure 2. Numbers of feeding pauses (± SE) by parasitised and unparasitised larvae (Treatment main effect: $F = 2.319$, $p = 0.150$; Time main effect: $F = 84.333$, $p < 0.001$; Treatment * Time: $F = 2.290$, $p = 0.147$).

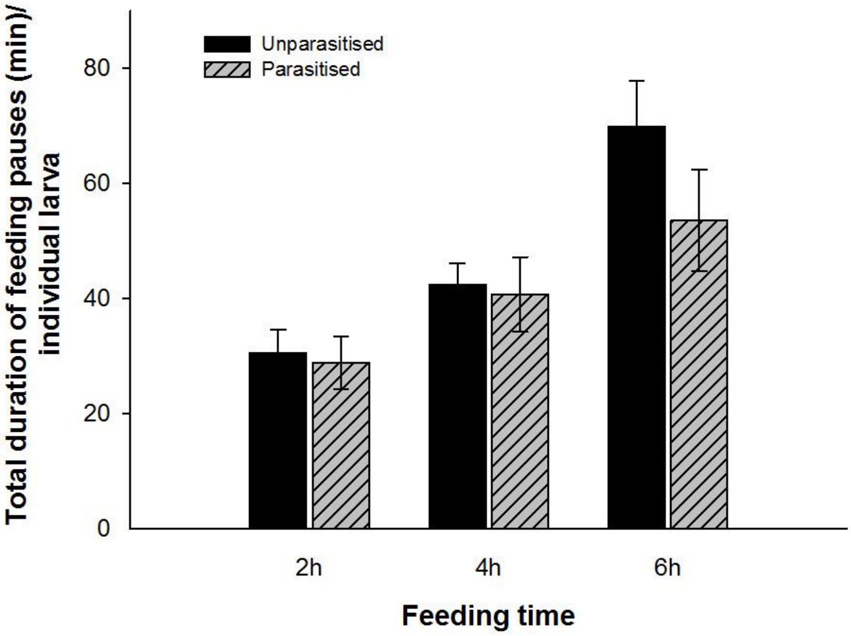

Supplementary Figure 3. Total duration of feeding pauses (± SE) by parasitised and unparasitised larvae (Treatment main effect: $F = 0.716$, $p = 0.412$; Time main effect: $F = 39.061$, $p < 0.001$; Treatment * Time: $F = 2.615$, $p = 0.120$).

Analysis of feeding behaviour on excised leaves revealed that parasitism did not affect the time spent feeding. The total duration of pauses in feeding did not differ between treatments (Supplementary Figure 2). Similarly, the numbers of feeding pauses did not differ between parasitised and unparasitised larvae (Supplementary Figure 3).
